# The Effect of the Chamber-Filling Ratio in Vibratory Shot Peening on Selected Surface Layer Properties of 30HGSA

**DOI:** 10.3390/ma18010008

**Published:** 2024-12-24

**Authors:** Agnieszka Skoczylas, Kazimierz Zaleski

**Affiliations:** Department of Production Engineering, Faculty of Mechanical Engineering, Lublin University of Technology, 36 Nadbystrzycka, 20-618 Lublin, Poland; k.zaleski@pollub.pl

**Keywords:** vibratory shot peening, surface roughness, surface topography, microhardness, residual stress, ball diameter, work-chamber-filling ratio

## Abstract

This study investigated the influence of the filling ratio of the working chamber and ball diameter in vibratory shot peening (VSP) on select properties of the surface layer. The tested material was 30HGSA steel, which is effectively used in the aviation industry. The following were analyzed: the surface roughness parameters, the shape of the Abbott–Firestone curve, the bearing area ratio Smr_(c=50%)_, the microhardness distribution, the microhardness on the surface, and the residual stress σ on the surface. A change in the ratio of peaks and valleys in the maximum height of the profile was observed. After VSP, the valleys were dominant over the peaks. The most favorable values of the analyzed roughness parameters (Sz, Sp, and Sv) were obtained for d = 9.4 mm and k_d_ = 33%. The bearing area ratio Smr_(c=50%)_ was approximately 50 times higher than before VSP (the most favorable for d = 9.4 mm and k_d_ = 33%). The largest thickness of the strengthened layer of 200 μm and the greatest increase in the microhardness equal to ΔHV 0.05 = 109 were obtained after VSP was conducted using the ball diameter d = 14.3 mm k_d_ = 33%. Regardless of the VSP conditions, the absolute value of compressive stresses increased; the highest σ stresses were obtained for d = 3.0 mm and k_d_ = 33%, and they were 88% higher than before the treatment. It was concluded that the recommended chamber-filling ratio for beneficial properties is k_d_ = 33%.

## 1. Introduction

The operational durability of machine components depends, to a large extent, on the condition of their surface layer formed in the manufacture process [1,2]. The condition of the surface layer, which improves the functional properties of machine components, can be shaped using various methods [3,4]. One of the most important functional properties of machine components is fatigue strength, as evidenced by experimental research and numerical modeling [5,6]. A processing method that has a positive effect on the service life of the workpieces, and especially on their fatigue resistance, is shot peening [7,8]. The strengthening of the surface layer occurs as a result of impacts of peening elements, such as balls or shots, on the treated surface [9,10]. A shot-peening process carried out in a way that ensures the guidance of peening elements, making it possible to maintain a constant distance between the traces of successive impacts, is called concentrated peening, while shot peening with free peening elements randomly hitting the treated surface is known as dispersed peening [11]. The dispersed shot peening method is more widely used as it enables the strengthening of objects with complex shapes [12,13].

Shot peening changes the surface texture of the workpieces. Depending on the processed material and the technological conditions of shot peening, the roughness of the processed surface may vary within a very wide range. A study by F. Yazdani et al. demonstrated that the AISI 316L austenitic stainless-steel shot peened with cast steel balls of a diameter of 580 µm had the surface roughness of Ra = 0.045 µm [14]. On the other hand, a study by H. Chen et al. showed that the value of the Ra surface roughness parameter of shot-peened high-manganese steel varied from a dozen to about 120 µm, depending on the shot-peening conditions [15].

The impact of shot-peening elements, as well as the static impact of burnishing elements, cause the microhardness of the surface layer of workpieces to increase. After the shot peening of XCrNiNb1810 stainless steel, an increase in the microhardness of the surface layer was observed at a depth of approximately 200 ÷ 400 µm, depending on the shot-peening parameters [16]. Skoczylas and Kłonica [17] showed that the microhardness of the surface layer also depended on the properties of the cutting fluid used in the strengthening process. Similarly to burnishing, the microhardness of the surface layer after shot peening is usually measured on microsections by the Vickers method [18,19]. The use of this method involves the destruction of the tested element. The strengthening of the surface layer can be assessed via positron annihilation index tests, which are non-destructive tests. Also, the recurrence analysis [20] allows for the evaluation of defects created during machining [21]. Another study [22] showed that the increase in the degree of strengthening and the depth of hardening of the surface layer resulted in longer positron lifetime (τ_mean_).

An important property of the surface layer is the residual stress generated during the machining process because it has a significant impact on the fatigue strength of manufactured components. A study by W. Qian et al. showed that shot peening induced compressive residual stresses in the surface layer of 20MnTiB steel specimens, the absolute value of which could be as high as 730 MPa [23]. T. Gundgire et al. studied the effects of heat treatment at different temperatures and severe shot peening on the residual stresses in 316 L steel elements [24].

One variation in shot peening is vibratory shot peening, a process which is conducted in chambers containing workpieces and peening elements (e.g., steel balls or ceramic shapes). The working chamber is set in an oscillating motion, which causes the shot peening elements to hit the workpieces, leading to the strengthening of the surface layer of these workpieces [25,26]. The effects of shot peening depend on the technological parameters of this process. T. Das et al. analyzed the effect of vibratory shot-peening time on the microstructure, microhardness, and tribological properties of AISI 1020 steel [27]. J. Matuszak studied the effects of vibrator amplitude and vibratory peening time on the surface roughness and microhardness of the surface layer in Ti6Al4V titanium alloy samples [28]. The vibratory shot-peening process of Ti6Al4V titanium alloy and carburized steel E-16NiCrMo13 was studied by L. Canals et al., who evaluated the influence of vibration frequency, machining media mass, and process duration on the surface roughness, residual stresses, and hardness of the workpieces [29]. Another study [30] investigated the effect of the diameter of balls used as the machining medium in vibratory shot peening on the surface texture of samples cut with a laser and abrasive water jet. A significant reduction in strengthening machining time was achieved by A. Gopinath et al., who applied a smart vibratory peening system using 4 mm diameter balls as the machining medium [31]. The changes in the surface layer properties induced by vibratory shot peening also depend on the processed material, as confirmed by a study investigating the effect of shot peening time on the residual stress distribution and positron lifetime in samples made of C45 steel, 7075 aluminum alloy, and Ti6Al2Mo2Cr titanium alloy [32]. D. Kumar et al. compared the effect of shot peening and vibro-peening on the surface texture, microstructure and residual stresses of a nickel alloy [33]. Favorable changes in the surface layer properties and increased fatigue life of C45 steel samples were obtained as a result of vibratory–rotational shot peening, which consisted of imparting rotational motion to the shot-peened objects in a vibration chamber [34]. The strengths of vibratory shot peening include the possibility of obtaining a lower surface roughness than after shot peening [29], which limits the formation of microcracks on the treated surface, and the development of compressive residual stresses, which occur at greater depths than after SP [29]. Tribological properties also improved [27]. The favorable properties of the surface layer after VSP, primarily the state of residual stresses, allow for improved fatigue performance [35,36]. The results from the work of [37] showed that the use of vibration finishing after shot peening allowed for an increase in the fatigue limit both at room temperature and at elevated temperatures. The analysis of vibratory shot peening processes can be improved by numerical modeling of these processes [38,39].

Previous studies on vibratory shot peening focused on the influence of intensity vibratory shot peening [33], time [28,29,36], frequency [29], size of shot penning medium [29,37], type of shot peening medium [39], and amplitude value on the selected properties of the surface layer. Our own work [25,30] showed the influence of vibratory shot peening time and ball diameter on surface roughness, surface topography, and microhardness. There is a lack of work on vibratory shot peening that show the influence of the quantity of the shot penning medium (expressed by the filling ratio of the working chamber) on the VSP effect. The aim of this work was to experimentally study the influence of the chamber filling ratio in vibratory shot peening on the selected properties of the surface layer of the workpieces.

## 2. Materials and Methods

The vibratory shot peening process was carried out on 4x15x100 rectangular specimens made of 30HGSA alloy steel. This steel grade is used for high-strength parts, e.g., gears, spindles, and levers, and in the manufacture of airframes, piston engines, and aircraft engines [40]. The 30HGSA steel is used to manufacture components of the wing strut joint of an aircraft [41] and solid propellant rocket motor nozzles [42]. The chemical composition and selected properties of the 30HGSA alloy steel grade are listed in Table 1.

**Figure 1:**
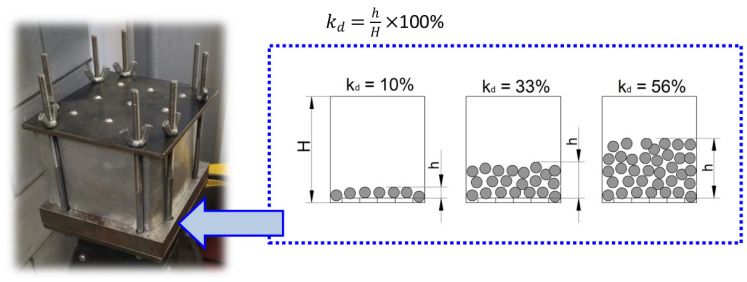
Working chamber used in a vibratory shot-peening process and a graphical representation of the chamber filling ratio (H—height of the working chamber, h—charge height).

Grinding was used as a pretreatment. The grinding process was carried out on an Okamoto ACC 64CA-iQ CNC (Okamoto, Gobara, Japan) surface and profile grinder. An emulsion was used as the cutting fluid. A Norton Saint-Gobain grinding wheel with precious electrocorundum grains, technical characteristics of 38A60M7VS3 and dimensions of 400 × 50 mm, was used for grinding. The following technological parameters were used: cutting speed v_c_ = 20 m/s; depth at a single pass a_p_ = 0.1 mm; number of flashing passes: 2. The operation was carried out on an SPC grinder using low feed and abundant cooling. After the pretreatment, the samples were subjected to vibratory shot peening (VSP). The vibratory shot-peening process was performed on a specially designed stand (Figure 1), which consisted of a mechanical–kinematic vibrator and the working chamber with a height of H = 90 mm. It is not a commercial device and is characterized by the following parameters: 1.1kW electric motor, with a rated speed of 1400 rev./min, and belt transmission with replaceable pulleys. At the bottom of the working chamber were mounted 30HGSA samples, which were impacted during the tests by shot-peening elements with a variable diameter d, the so-called charge. The diameter balls were made from 100Cr6 bearing steel. The variable factors were as follows: the diameter d of the ball (the so-called working medium) and the ratio of filling the working chamber, k_d_ (Figure 1). The chamber filling ratio was determined using formula (1). The k_d_ ratio is the percentage of the shot peening elements (charge height—h) in relation to the height of the working chamber H. The working chamber has a cuboid shape. The experiment was carried out according to the plan shown in Table 2; in the same table, the constant condition VSP was presented. The parameters of the vibratory shot-peening process were selected based on our previous research on vibratory shot peening [25,31].

(1) $$k_{d}=\frac{h}{H}\times 100\%$$

where

H—height of the working chamber;
h—charge height.

According to [44,45], surface topography measurements were made, and 3D surface roughness parameters were determined. The measurements were performed on a T800 RC 120-400 (Jenoptik, Villingen-Schwenningen, Germany) device from Hommel Etamic, equipped with a TKU300 measuring tip that was fitted with a measuring needle with a radius of r_k_ = 5 μm. The scanned area had the following dimensions: 4.8 × 4.8 mm. The following roughness parameters were analyzed: arithmetical height of the surface—Sa, maximum height of the surface—Sz, maximum peak height of the surface—Sp, maximum pit height of the surface—Sv, and extreme height of the peaks—Sxp. For the analyzed surface, the material ratio curve and the bearing area ratio (Smr) were determined at a cut-off level of c = 50%.

Leco’s LM 700at (Leco, St. Joseph, MI, USA) microhardness tester was used to measure the microhardness on the surface and cross-microsections after standard processing. The Vickers method was used for measurement, using a penetrator load of 50 g (HV 0.05) and 100 g (HV 0.1).

A portable diffractometer Theta-Theta EDGE was used to measure residual stresses. The measurements were made in one direction. A chrome lamp was used as the XRD beam source, and a vanadium filter was utilized. A 0.5 mm collimator was used. The XRD beam exposure time was 30 s.

**Figure 2:**
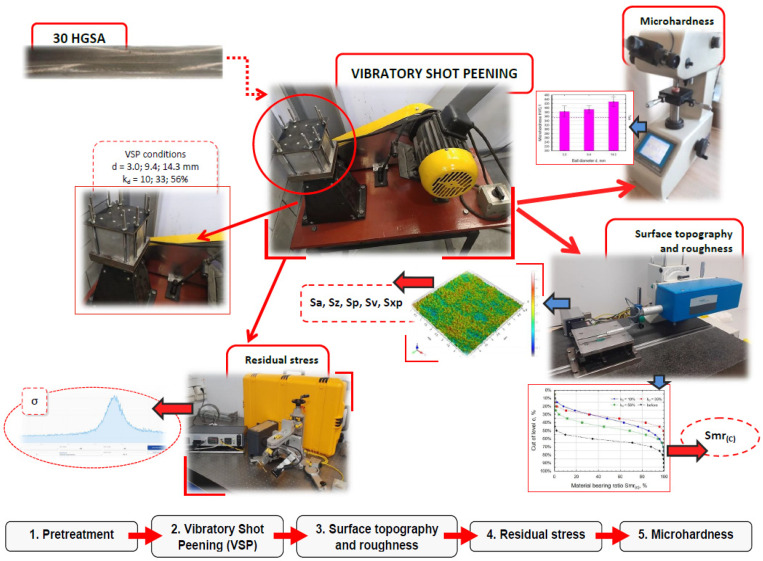
Research methodology, stands, and flowchart of the work carried out.

Figure 2 shows the test stands and methods of measurements made on 30HGSA steel samples before and after the vibratory shot-peening process.

## 3. Results and Discussion

### 3.1. Surface Topography

**Figure 3:**
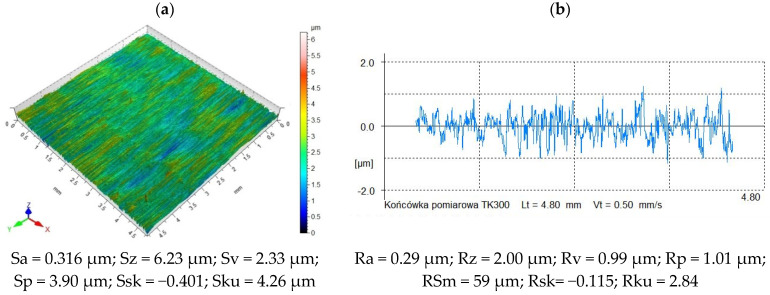
Surface topography (**a**) and profilogram of a sample (**b**) after grinding (pretreatment).

Figure 3a shows the surface topography of the sample before vibratory shot peening (after grinding). It can be observed that the micro-irregularities have a unidirectional pattern, which is a result of the pretreatment (grinding). Single peaks are visible, which is most likely a surface defect. The resulting micro-irregularities are close to each other, which is confirmed by the surface profilogram (Figure 3b). The average height of the micro-irregularities ranges from 1 μm to 2.5 μm. Peaks are dominant in the total height of surface irregularities. The Sp parameter is 67% higher than the Sv parameter, which results from the mechanics of the grinding process. The peaks of the micro-irregularities are strongly plastically deformed, which indicates material flow.

**Figure 4:**
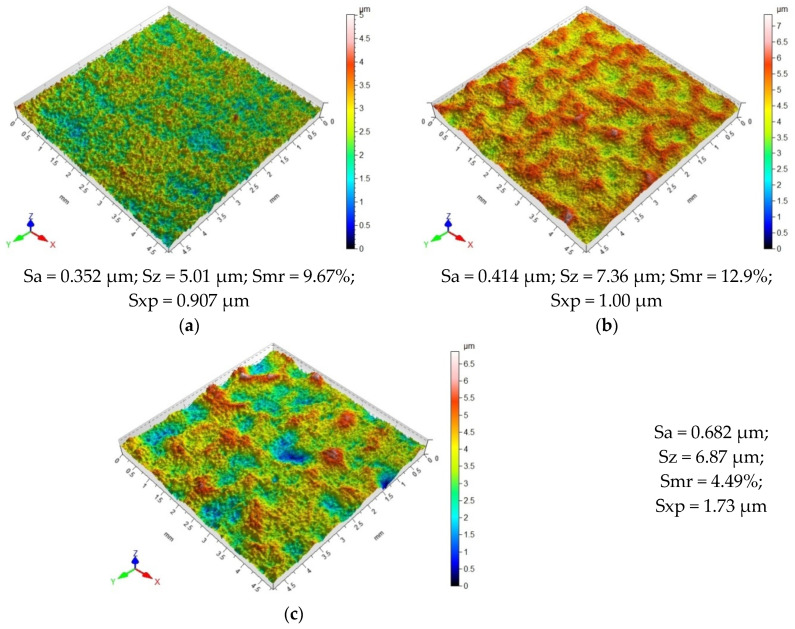
Surface topography after vibratory shot peening conducted with different ball diameters d: (**a**) d = 3.0 mm; (**b**) d = 9.4 mm; (**c**) d = 14.3 mm.

**Figure 5:**
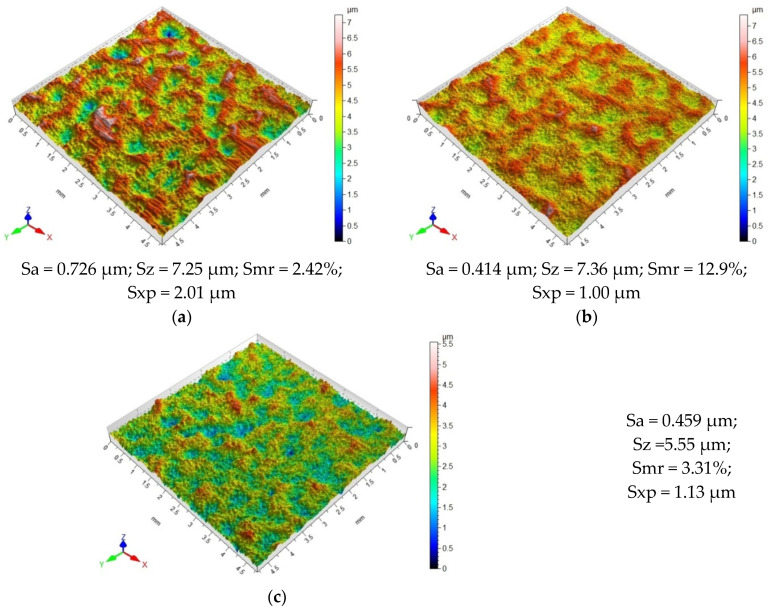
Effect of chamber-filling ratio on the surface topography of vibratory shot-peened objects: (**a**) k_d_ = 10%; (**b**) k_d_ = 33%; (**c**) k_d_ = 56%.

The VSP process changes the surface topography of the samples. For all vibratory shot-peening conditions, different patterns of micro-irregularities were obtained compared to that obtained after the pretreatment. The effects of impacts of the shot peening elements in the form of numerous peaks and valleys are visible on the surface (Figure 4 and Figure 5). Hitting the surface, the balls crush the peaks of the micro-irregularities that were formed after grinding. An analysis of the obtained surface topography as a function of the applied vibratory shot peening conditions reveals slight differences in the resulting patterns of micro-irregularities. An increase in the diameter of the balls used for VSP causes the marks of greater width to be “punched” on the sample surface and areas with a greater degree of deformation appear (Figure 4). As the diameter of the balls used for VSP is increased, the extreme peak height (parameter Sxp) increases, which may indicate a significant degree of deformation of the shot-peened surface. For the surfaces after VSP was conducted using the balls with d = 14.3 mm, numerous peaks of considerable height were observed, which results from the impact of heavier balls on the sample surface. Compared to the surface obtained after VSP conducted with d = 3 mm and d = 9.4 mm, this surface is characterized by the presence of significant micro-irregularities.

A comparison of the surface topography after VSP was conducted with different medium quantities (Figure 5) showed that for the shot-peened sample using a ratio of k_d_ = 10%, there are numerous valleys and areas of significant deformation on the surface. This is most likely due to the fact that the working chamber contained a small number of balls that repeatedly hit the same spot on the sample surface. In the chamber space, there is a lower probability for the mutual collision of the balls, as all of the impact energy is transferred to the shot-peened element. For the sample shot peened with k_d_ = 10%, the highest parameter Sxp of 2.01 μm was also obtained. An increase in the chamber-filling ratio to k_d_ = 33% caused the peaks of micro-irregularities to be cut off, which resulted in an increase in the bearing area ratio Smr. Unfortunately, a further increase in the k_d_ ratio to a value of 56% caused a decrease in the bearing area ratio Smr, which may indicate the formation of single sharp peaks resulting from the deformation of the shot-peened surface.

### 3.2. Surface Roughness

**Figure 6:**
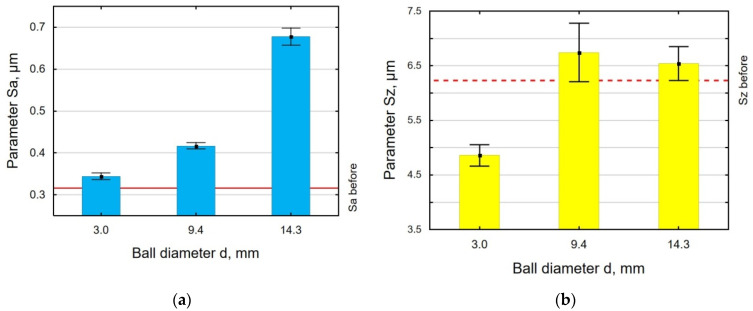
Effect of ball diameter in VSP of 30HGSA steel on the surface roughness parameters Sa (**a**) and Sz (**b**).

**Figure 7:**
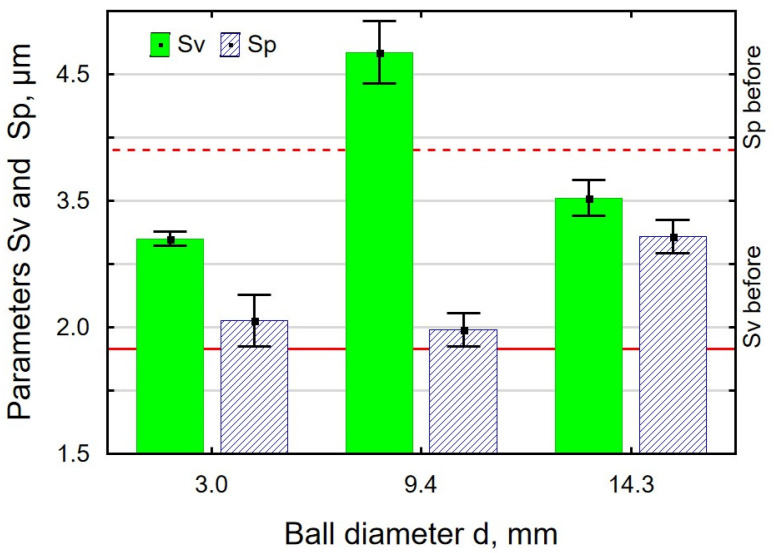
Effect of ball diameter in VSP of 30HGSA steel on the surface roughness parameters Sv and Sp.

Figure 6 and Figure 7 show the surface condition of 30HGSA steel samples subjected to vibratory shot peening using balls of different diameters. An analysis of the results demonstrated that, regardless of the ball diameter used, the Sa parameter is higher than before the treatment (Figure 6a). An increase in the ball diameter causes a decrease in the number of impacts per unit, which leads to an increase in surface roughness. The use of larger diameter balls also means a larger mass of the charge, which translates into greater impact energy. The largest increase was obtained for d = 14.3 mm, and the Sa parameter was 115% greater in relation to the value after grinding. The use of small diameter balls caused the maximum height to be smaller than before VSP, while for other ball diameters, it was larger (Figure 6b). Interesting results were obtained for the height parameters Sp and Sv (Figure 7) as a function of the ball diameters. First of all, there was a reconstruction of the ratio of peaks and valleys in the maximum surface height. After vibratory shot peening, the valleys were dominant, whereas before VSP, the peaks were dominant. For all ball diameters, the maximum peak height of the surface is smaller than after grinding, which confirms the phenomenon of crushing the micro-irregularities peaks after the pretreatment. The lowest value of the Sp parameter was obtained after VSP was conducted using the balls with a diameter of d = 9.4 mm, which confirms the occurrence of a mechanism for “smoothing out” micro-irregularities. As for the maximum pit height, the Sv parameter values are higher after VSP than after grinding. This means that pits are punched into the surface and thus can be potential lubrication pockets when making VSP-machined parts.

**Figure 8:**
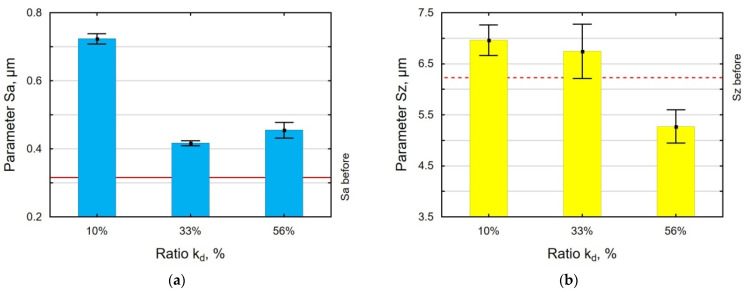
Effect of the k_d_ ratio in VSP of 30HGSA steel on the surface roughness parameters Sa (**a**) and Sz (**b**).

**Figure 9:**
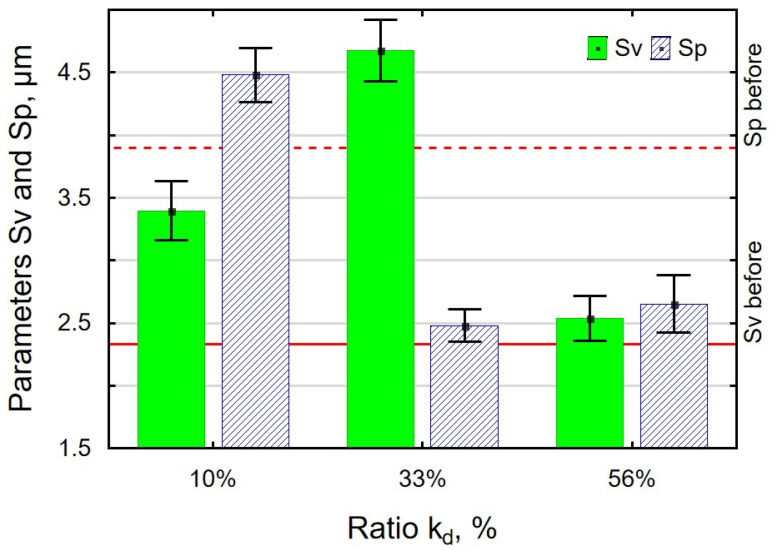
Effect of the k_d_ ratio in VSP of 30HGSA steel on the surface roughness parameters Sv and Sp.

An analysis of the effect of the work-chamber-filling ratio k_d_ on the parameters Sa, Sz (Figure 8), Sp, and Sv (Figure 9) revealed that the VSP process conducted with k_d_ = 10% resulted in all parameters having greater values than after grinding. An insufficient number of balls in the working chamber leads to an incomplete deformation of micro-irregularities. An interesting result is that the values of Sp and Sv obtained for k_d_ = 56% are similar, which means that the mechanism of knocking out the pits and cutting off the peaks occurs with the same frequency, which completely differs for k_d_ = 33%.

A comparison of the surface roughness parameters for vibratory shot-peened 30HGSA steel demonstrated that for most conditions, they were higher than the results obtained in another study [7] where Sa = 0.489 μm, but they were lower than the values obtained after the VSP of elements cut with laser and water abrasive jet presented in our previous research [30]. The difference from the results reported in [30] is due to the ball diameter d, whereas in the earlier study, the 3D surface roughness parameters would decrease with increasing ball diameter d. This is most likely due to the type of pretreatment. It was also noted that the surface roughness parameters, Sa and Sz, depended on the vibratory shot-peening conditions, while another study [29] showed that the process parameters had little effect on surface roughness.

**Figure 10:**
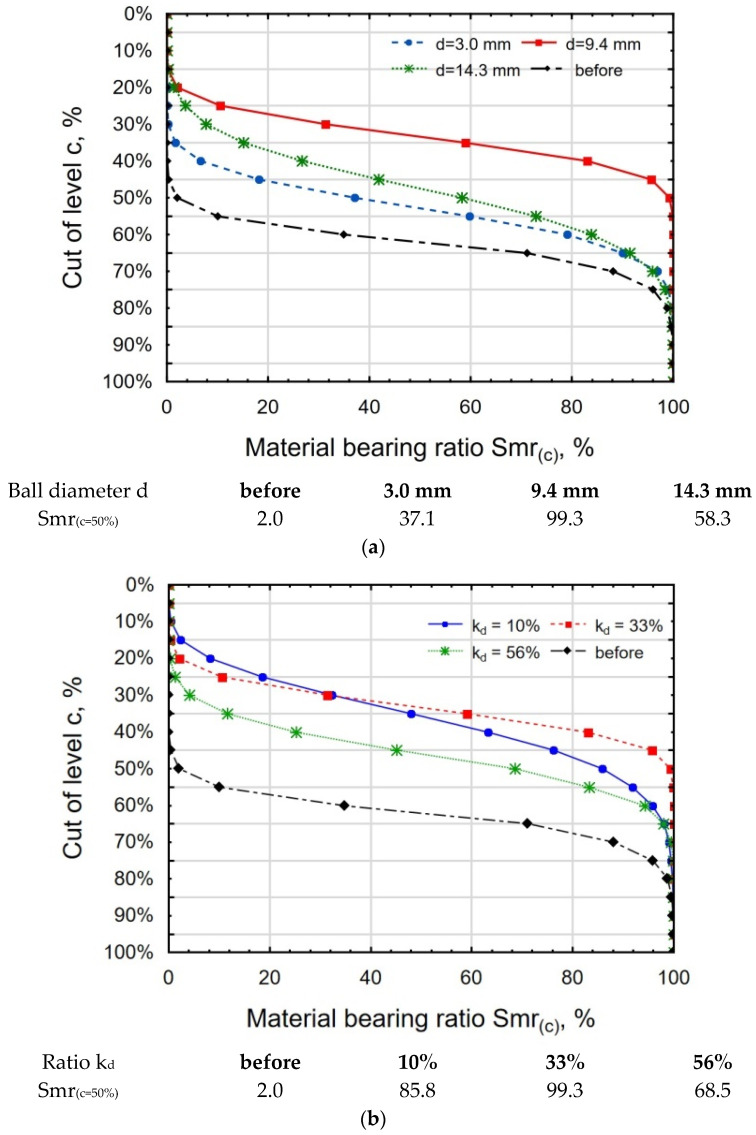
Abbott–Firestone curve for 30HGSA steel samples subjected to vibratory shot peening with variable ball diameter d (**a**) and variable k_d_ ratio (**b**).

The shape of the material ratio curve (Abbott–Firestone curve) and the value of Smr_(c=50%)_ confirmed the favorable changes induced by the height of the micro-irregularities in vibratory shot peening (Figure 10). The material surface ratio of the AF curve after grinding to the 50% cut-off level oscillates around 0%. The shape of the curve resembles a degressive curve. After VSP was conducted using the balls with d = 3.0 mm and d = 14.3 mm, the shapes of the curves were similar, resembling a degressive–progressive curve. The AF curve for the balls with d = 9.4 mm had a “wider” ridge, which can be related to the values of the parameters Sz and Sp (Figure 10a). An analysis of the effect of the k_d_ ratio on the shape of the AFC curve (Figure 10b) revealed that the flattest curve was obtained after a vibratory shot-peening process was conducted with k_d_ = 33%.

The obtained shape of the AF curve and the value of the bearing area ratio are similar to the results obtained for the Ti6Al4V titanium alloy after vibratory shot peening [28].

### 3.3. Microhardness and Residual Stress

**Figure 11:**
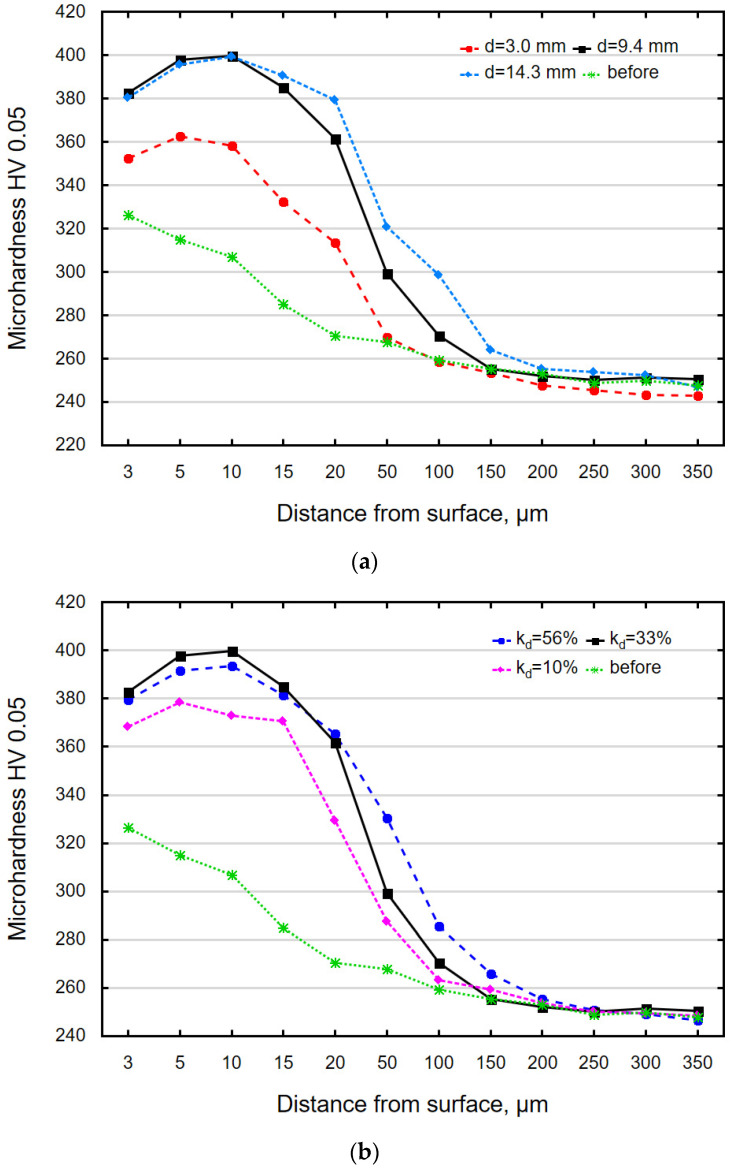
Microhardness distribution as a function of the distance from the surface for samples after: (**a**) vibratory shot peening with variable diameter d and (**b**) vibratory shot peening with variable k_d_.

Figure 11 shows the distribution of microhardness as a function of the distance from the surface for the ground sample and the sample after VSP. All samples after vibratory shot peening exhibit a microhardness distribution that is typical of vibratory shot-peened elements [25]. The differences in the distribution occur only in the depth of maximum microhardness and the width of VSP impact zone. Grinding as the pretreatment caused an increase in microhardness. The greatest microhardness increase occurred just below the surface (at a depth of 3 μm), and the thickness of the strengthened layer was about 20 μm. The balls hitting the machined surface caused plastic deformation, which led to an increase in the dislocation density. According to the dislocation theory, the resulting defects (dislocations) moved and stopped when they met other dislocations. The phenomenon of dislocation “locking” took place, which, consequently, led to an increase in microhardness. The highest microhardness and the greatest thickness of the strengthened layer were obtained after vibratory shot peening was conducted using balls with d = 14.3 mm. The microhardness increase was ΔHV 0.05 = 109 (at a depth of 20 μm), while the thickness of the strengthened layer was 150 μm, which is the same as the results reported in [33]. The microhardness distribution after vibratory shot peening conducted using the balls with d = 14.3 mm was more “stretched” than for the d = 9.4 mm balls and was also characterized by greater microhardness values at greater depths from the surface. The obtained thickness of the strengthened layer was greater than that reported in the previous study investigating the VSP of elements after water-jet cutting [30], which is most likely due to the initial state of the shot-peened elements.

An analysis of the effect of the k_d_ ratio (Figure 11b) on the microhardness distribution demonstrated that regardless of the k_d_ value, the distribution was similar, with the differences occurring only in the maximum microhardness increase (ΔHV) and the depth of occurrence of the maximum values. The highest microhardness increase was obtained for k_d_ = 33%, while the widest VSP impact zone was obtained for k_d_ = 56%. This means that the use of too many balls in VSP only affects the thickness of the strengthened layer. This should be explained by the fact that a larger number of balls in VSP means a larger mass of the charge, which leads to greater impact energy and thus contributes to increased thickness of the strengthened layer. On the other hand, the quantity of charge limits the height from which the balls hit the surface of the object. The impact energy is not transferred to the object; it is absorbed either by other balls or by the walls of the working chamber, which reduces the microhardness increase.

**Figure 12:**
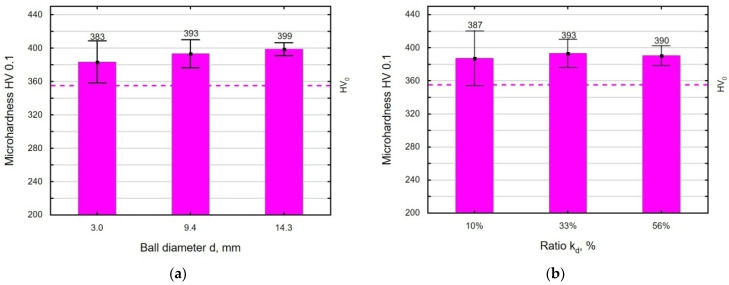
Effect of the ball diameter d (**a**) and the k_d_ ratio (**b**) on microhardness.

The VSP-induced changes in microhardness were also confirmed by measurements taken on the surface (Figure 12). The highest increase in the microhardness was obtained for the sample that was shot-peened using the balls with d = 14.3 mm and the working chamber filling ratio k_d_ = 33%. The results obtained from the surface microhardness measurements are identical to the microhardness distribution. The average increase in the microhardness on the surface for the variable diameter d is 10.3 ± 2.1 HV0.1, while for the variable k_d_, it is 9.9 ± 0.85 HV0.1, which is lower than for the E-16NiCrMo13 steel and Ti6Al4V titanium alloy after VSP [29].

**Figure 13:**
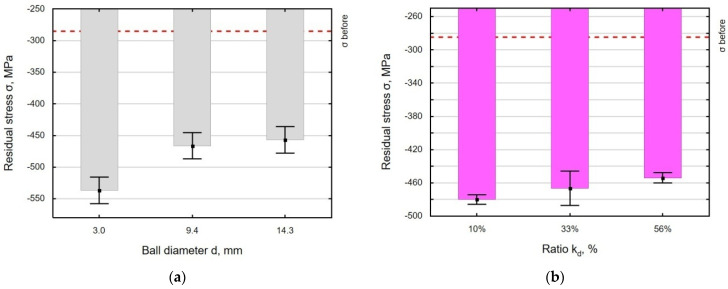
Effect of the ball diameter d (**a**) and the k_d_ ratio (**b**) on residual stresses.

The strain energy generated by the impact of the balls on the workpiece contributes to an increase in the absolute value of the compressive residual stress (Figure 13). After grinding, the compressive stresses in the surface layer are equal to −285 MPa. The maximum residual stresses were obtained for the balls with a diameter of d = 3.0 mm, and they were 88% greater than the pre-stress values (Figure 13a). It should also be noted that for ball diameters ranging from 9.4 mm to 14.3 mm, there is no noticeable change in the stress values. When analyzing the effect of the k_d_ ratio on the stress value, it should be noted that there are no clear changes in the residual stress state in the entire range (Figure 13b). The lack of clear changes in the residual stress state as a function of the k_d_ ratio is related to microhardness, where no significant differences were observed either.

A comparison of the residual stresses after VSP with the values reported in previous studies revealed that the obtained stress values were lower than those reported in [29].

## 4. Conclusions and Summary

This study investigated the vibratory shot-peening process of 30HGSA steel samples that was conducted with variable parameters (ball diameter d and chamber filling ratio k_d_). Based on the obtained results, the following can be concluded:

1. The VSP process changes the surface topography of the samples, producing a surface with a point-like pattern of micro-irregularities.
2. For all vibratory shot-peening conditions, the surface roughness parameter Sa is greater than before treatment. On the other hand, the use of the following VSP process parameters, d = 3 mm and k_d_ = 33% and d = 9.4 mm and k_d_ = 56%, results in a reduction in the Sz parameter with respect to its value after pretreatment.
3. After vibratory shot peening, the ratio of peaks and valleys in the maximum surface height (parameter Sz) changed. Specifically, the valleys were dominant after VSP, whereas the peaks were dominant prior to the treatment.
4. Regardless of the ball diameter d, the use of the chamber filling ratios k_d_ = 33% and k_d_ = 56% after VSP yielded lower values of the Sp parameter compared to the values after grinding. For all VSP conditions analyzed in the experiment, the roughness parameter Sv was greater than before the treatment. This means that valleys were formed on the surface, which could be lubrication pockets for oil.
5. The material ratio curve after VSP changed, acquiring a degressive–progressive shape. The parameter Smr_(c=50%)_ increased, which may suggest an increase in the abrasive wear resistance of the VSP-treated components.
6. The pretreatment caused an increase in the microhardness to ΔHV 0.05 ≈ 59, while the depth of the strengthened layer was approximately 50 μm.
7. The width of the VSP impact zone ranges from 50 μm to 200 μm. The widest VSP impact zone was obtained for d = 14.3 mm and k_d_ = 33% and k_d_ = 56% and d = 9.4 mm. The largest increase in microhardness was obtained with d = 14.3 mm and k_d_ = 33%.
8. After VSP, compressive residual stresses were generated for all vibratory shot peening conditions. The highest residual stresses were obtained for d = 3 mm and k_d_ = 33% and were 88% higher compared to their values after grinding.
9. The use of a small amount of charge (k_d_ = 10%) caused the deterioration of the surface quality and a small increase in microhardness.

The results of this study, which investigated select properties of the surface layer of 30HGS steel after vibratory shot peening, demonstrated that for beneficial effects, the shot-peening process should be conducted with a ball diameter of d = 9.4 mm and a chamber filling ratio of k_d_ = 33%.

## Figures and Tables

**Table 1:** Chemical composition and selected properties of 30HGSA steel [43].

Chemical Composition, %							
C	Si	Mn	Cr	Ni	P	S	Fe
0.28–0.34	0.9–1.2	0.8–1,1	0.8–1.1	<0.3	<0.025	<0.025	rest
Tensile strength Rm, MPa				>1080			
Conventional yield point Rp_0.2_, MPa				377			
Unit elongation A, %				>9			
Vickers hardness				<241			

**Table 2:** Variable parameters of a vibratory shot-peening process.

No.	Vibrator Frequency ν, Hz	Vibrator Amplitude a, mm	Time VSP t, min	Ball Diameter d, mm	Ratio k_d_, %
1.	23	10.3	5.0	3.0	33
2.				9.4	
3.				14.3	
4.				9.4	10
5.					56

## Data Availability

The data presented in this study are available on request from the corresponding author due to the data also form part of an ongoing study at this time.
